# Periodontal Management in Periodontally Healthy Orthodontic Patients with Fixed Appliances: An Umbrella Review of Self-Care Instructions and Evidence-Based Recommendations

**DOI:** 10.3390/dj11020035

**Published:** 2023-01-31

**Authors:** Federica Di Spirito, Alessandra Amato, Maria Pia Di Palo, Davide Cannatà, Francesco Giordano, Francesco D’Ambrosio, Stefano Martina

**Affiliations:** 1 Department of Medicine, Surgery and Dentistry, University of Salerno, 84081 Salerno, Italy; 2 Department of Neuroscience, Reproductive Science and Dentistry, University of Naples Federico II, 80138 Naples, Italy

**Keywords:** periodontal management, periodontal health, orthodontic treatment, fixed appliances, biofilm control, gingivitis, home-care, self-care

## Abstract

The present umbrella review aimed to characterize periodontal self-care instructions, prescriptions, and motivational methods; evaluate the associated periodontal outcomes; and provide integrated, evidence-based recommendations for periodontal self-care in periodontally healthy orthodontic patients with fixed appliances. The presently applied study protocol was developed in advance, compliant with the PRISMA statement, and registered on PROSPERO (CRD42022367204). Systematic reviews published in English without date restrictions were electronically searched until 21 November 2022 across the PROSPERO Register and Cochrane Library, Web of Science (Core Collection), Scopus, and MED-LINE/PubMed databases. The study quality assessment was conducted through the AMSTAR 2 tool. Seventeen systematic reviews were included. Powered and manual toothbrushes showed no significant differences in biofilm accumulation, although some evidence revealed significant improvements in inflammatory, bleeding, and periodontal pocket depth values in the short term with powered toothbrushes. Chlorhexidine mouthwashes, but no gels, varnishes, or pastes, controlled better biofilm accumulation and gingival inflammation as adjuncts to toothbrushing, although only for a limited period. Organic products, such as aloe vera and chamomile, proved their antimicrobial properties, and herbal-based mouthwashes seemed comparable to CHX without its side effects. Motivational methods also showed beneficial effects on periodontal biofilm control and inflammation, while no evidence supported probiotics administration.

## 1. Introduction

Periodontal health is defined by the absence of microscopically and macroscopically detectable signs of inflammation interfering with periodontal physiology [1]. Given the well-known role of biofilm accumulation in gingivitis and periodontitis onset and development [2], the potential periodontal self-care and oral hygiene procedures are essential for maintaining periodontal health, supported by regular check-ups and professional operative sessions [1,2].

Fixed orthodontic treatment provides tooth movement to correct dental malocclusion through appliances, such as orthodontic bands and brackets, bonded to the tooth surface, archwires, ligatures, and auxiliaries [3,4]. Fixed orthodontic appliances often complicate oral hygiene procedures [5] and facilitate biofilm accumulation on both teeth and appliance surfaces [6,7,8].

Indeed, biofilm control and clinical periodontal inflammatory parameters are generally worse in orthodontic patients with fixed appliances than in patients with removable appliances and non-orthodontic patients [8]. Indeed, patients with fixed orthodontic appliances often have difficulty maintaining good oral hygiene during treatment, negatively impacting periodontal health [9,10], thus negatively affecting periodontal health maintenance. In addition, since it has long been known that uncontrolled periodontal inflammation during orthodontic treatment precipitates periodontitis progression and tissue destruction [11,12,13], a complete diagnosis that takes into account both the orthodontic and periodontal needs as well as the achievement of periodontal stability before starting orthodontic treatment is strongly recommended [14].

Conversely, if periodontal biofilm and inflammation are adequately controlled, no long-term [15] detrimental effects on clinical [15,16] and microbial periodontal parameters are expected in periodontally healthy patients after the removal of orthodontic appliances [17,18,19]. Consequently, based on evidence supporting that periodontal health, with interconnected systemic relapses, crucially relies on patients’ self-care, several measures for effective and efficient mechanical and chemical biofilm control have been proposed to achieve and maintain healthy periodontal conditions in patients undergoing fixed orthodontic treatment [20,21].

Among these, manual toothbrushing as a daily routine is considered the primary healthy behavior intervention [22] in gingivitis prevention [23]. In addition, orthodontic toothbrushes, which are manual devices specifically designed to increase the contact area between the toothbrush’s bristles and the orthodontic appliance [24], may also be helpful [25].

Chlorhexidine, a broad-spectrum antiseptic, remains the gold standard for the chemical control of oral and periodontal biofilm [26], while other organic products with proven antimicrobial properties against oral bacterial species, such as aloe vera and herbal-based mouthwashes, are also available [27,28,29].

Moreover, probiotics, which are live microorganisms that provide health benefits to the host when administered in specific amounts [30], have been proven to disrupt the periodontal biofilm and modulate the host’s immune response [31,32], thus potentially improving biofilm control, reversing dysbiosis, and reducing periodontal inflammation [33].

Indirect interventions to prevent biofilm accumulation and reinforce patients’ motivation and literacy toward periodontal self-care in orthodontic patients with fixed appliances are also widely used [34].

Nevertheless, a comprehensive approach to effectively manage the periodontal health status in subjects undergoing fixed orthodontic treatment has not been defined yet.

Considering that lifelong periodontal self-care education, motivation, and guidance are prerequisites for achieving and maintaining healthy periodontal conditions [35], and that biofilm control may be challenging in orthodontic patients with fixed appliances [6,7,8], the present review aimed to characterize periodontal self-care instructions, prescriptions, and motivational methods; to evaluate and compare the associated periodontal outcomes; and to provide integrated evidence-based recommendations for periodontal self-care in periodontally healthy patients undergoing fixed orthodontic treatment.

## 2. Materials and Methods

### 2.1. Study Protocol

The present study protocol was registered in the PROSPERO International Prospective Register of Systematic Reviews (CRD42022367204), which was developed in accordance with the PRISMA (Preferred Reporting Items for Systematic Reviews and Meta-analyses) statement [36], before the literature search, data extraction, and analysis, with the research questions focusing on periodontal self-care instructions, prescriptions, and motivational methods [37].

The formulation of the study question, the definition of the search strategies, and the criteria for study selection were developed according to the PICO model [38]. The study question was “What is the current gold standard for home care instructions, prescriptions, and motivational methods in periodontally healthy orthodontic patients with fixed appliances?” focusing on the following:

P—Population: periodontally healthy orthodontic patients (without age or gender restrictions) with fixed (vestibular or lingual) appliances;

I—Intervention: periodontal self-care instructions, prescriptions, and motivational methods (any);

C—Comparison: no intervention, placebo, between different interventions;

O—Outcome(s): periodontal health status measured by periodontal indices (no self-report).

### 2.2. Search Strategy

**Figure 1:**
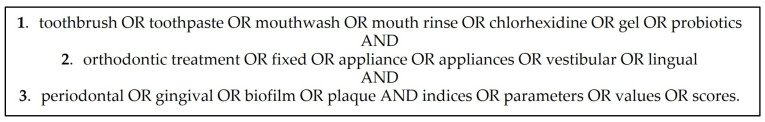
Keywords used for electronic cross-search databases.

Systematic reviews with or without a meta-analysis published in English without date restriction and related to periodontal self-care instructions, prescriptions, and motivational methods were searched electronically by two independent reviewers (F.D.S. and M.P.D.P.) through 21 November 2022, in the PROSPERO Registry and the Cochrane Library, Web of Science (Core Collection), Scopus, and MEDLINE/PubMed databases, combining the keywords illustrated in Figure 1 with Boolean operators, and applying the following filters: “Review (English)” and “refine: systematic review” in the Web of Science database; “Review (English)” in the Scopus database; “Systematic Review (English)” in the MEDLINE/PubMed database; “Keywords” and “Review” in the Cochrane Library; “Systematic review,” “Meta-analysis,” and “Completely published” in the PROSPERO register.

### 2.3. Study Selection and Eligibility Criteria

The collected citations were recorded, duplicates were eliminated using the reference management tool EndNoteTM (Clarivate), and the remaining titles were screened by two independent reviewers (F.D.S. and M.P.D.P.). The same two reviewers independently screened potentially relevant title-abstracts of systematic reviews with or without a meta-analysis.

Full texts of records that met the eligibility criteria and the ambiguous title-abstracts were obtained. No contact with the study authors was necessary because all full texts were available. The three authors independently reviewed the full texts (F.D.S., M.P.D.P., and D.C.). Any disagreement was resolved by discussion and consensus with a fourth author (F.D.A.) when necessary.

Inclusion criteria were as follows: systematic reviews with or without a meta-analysis published in English regarding periodontal self-care instructions, prescriptions, and motivational methods (of any type) in periodontally healthy orthodontic patients with fixed appliances. No restrictions were placed on the publication date or type of instructions, prescriptions, and motivational methods.

Exclusion criteria were as follows: duplicate records, commentaries, and editorials; and in vitro, preclinical, and clinical studies involving subjects with periodontitis, oral, and dental infections [39,40]; and patients undergoing orthodontic treatment with removable appliances; self-reports and concerns about periodontal status reported in the systematic reviews were not considered.

### 2.4. Data Extraction and Collection

Data were extracted independently by three authors (F.D.S., M.P.D.P., and D.C.) using a standardized data extraction form developed along the lines of the models proposed for intervention reviews of RCTs and non-RCTs [37]; a fourth author (F.D.A.) was consulted in case of disagreement.

From each systematic review with or without meta-analysis included in this review, the following data criteria were collected:

- first author, year, journal, funding, quality of the study;
- design and number of studies included in each review;
- sample size, gender ratio, and mean age of the study population of each systematic review;
- fixed orthodontic treatment performed: type and duration;
- periodontal self-care instructions, prescriptions, and motivational methods provided, and comparison(s), if applicable;
- evaluated clinical periodontal outcomes;
- statistically significant results and conclusion(s) of the study.

In detail, periodontal outcomes included clinical indices, such as clinical attachment loss (CAL), periodontal probing depth (PPD), bleeding on probing (BoP), gingival bleeding index (GBI), bleeding index (BI), gingival index (GI), modified gingival index (MGI), plaque index (PI), and others, as well as radiographic, crevicular, and any other parameters reported in the systematic reviews.

### 2.5. Data Synthesis

A narrative synthesis was conducted that focused on the population studied, the intervention, and the outcomes. Data from the included studies were qualitatively summarized by a descriptive statistical analysis using Microsoft Excel software 2019 (Microsoft Corporation, Redmond, WA, USA):

▪ to characterize periodontal self-care instructions, prescriptions, and motivational methods provided and comparison(s);
▪ to assess clinical periodontal outcomes in relation to the periodontal self-care instructions, prescriptions, and motivational methods provided;
▪ to compare clinical periodontal outcomes following the provision of the periodontal self-care instructions, prescriptions, and motivational methods compared to no intervention, to placebo, and each other.

### 2.6. Quality Assessment

The quality of the systematic reviews included was assessed using the Assessing the Methodological Quality of Systematic Reviews (AMSTAR) 2 tool, accessed online on 22 November 2022 (https://amstar.ca), which evaluated for quality the systematic reviews of randomized and/or non-randomized studies [41].

## 3. Results

### 3.1. Study Selection

The electronic search yielded 94 records from MEDLINE/PubMed, 79 from Scopus, 17 from the Cochrane Library, 176 from Web of Science (Core Collection), and 14 from the PROSPERO Registry, for a total of 380 records. Ninety-nine duplicate records were removed.

The 17 systematic reviews [27,28,29,34,42,43,44,45,46,47,48,49,50,51,52,53,54] included 145 randomized controlled trials (RCTs) [27,28,29,34,42,43,44,45,46,47,48,49,50,51,52,53,54], 7 non-RCTs [27,45,52], 3 controlled clinical trials (CCTs) [34,44,53], 3 quasi-experimental trials [49], 1 quasi-random trial [34], and 1 before/after study [53]. The extracted data are reported in Table 1.

The remaining 281 records were screened, of which 214 did not meet the eligibility criteria and were therefore excluded.

Of the remaining 67 articles, the full texts were read. No contact with the authors was required to obtain the full text or further information.

An additional 54 articles were excluded because they did not meet this study’s inclusion and exclusion criteria. Specifically: 28 studies involved subjects who did not undergo fixed orthodontic treatment; 6 did not evaluate periodontal parameters; 1 was not written in English; 1 did not apply an evaluable intervention; 1 included patients undergoing both fixed and mobile orthodontic treatment, and periodontal outcomes were not discernable; 1 systematic review did not include any study compliant with the eligibility criteria applied; 13 were narrative reviews.

Thus, 15 articles [27,28,29,42,43,44,45,46,47,48,49,50,51,52,53] from the electronic search were included in this systematic review.

Two additional studies compatible with the eligibility criteria [34,54] were found manually by reviewing the reference lists of the included articles.

**Figure 2:**
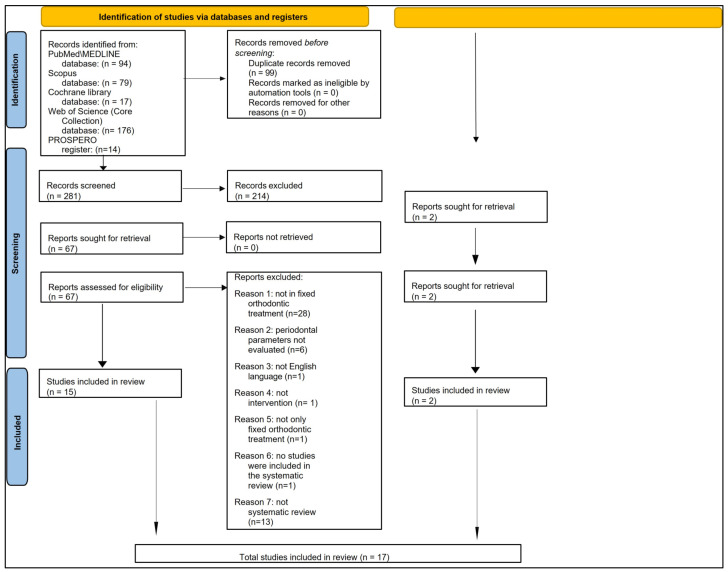
PRISMA 2020 flow diagram for new systematic reviews which included searches of databases, registers, and via other methods.

Figure 2 shows the flow diagram for the study selection, which included electronic searching databases, registries, and other methods.

Finally, 17 articles [27,28,29,34,42,43,44,45,46,47,48,49,50,51,52,53,54] were included in this systematic review.

### 3.2. Study Characteristics

Of the 17 systematic reviews [27,28,29,34,42,43,44,45,46,47,48,49,50,51,52,53,54] included in this study, 9 included a meta-analysis [34,42,43,45,46,47,48,49,54], and only 1 study [52] claimed to have received external funding. At the AMSTAR 2 quality assessment, twelve systematic reviews [27,28,29,34,43,44,45,46,49,50,52,53] were of critically low quality, and five were of low quality [42,47,48,51,54].

The total sample size was 7.547, although one study [53] did not report the number of subjects involved. Participants, 1.835 males and 2.818 females, corresponding to a ratio of M:F = 1:1.5, were between 8 and 64 years old, although gender was not specified for 2894 subjects, and mean age was hardly ever reported.

Gingival/periodontal health status was reported in only 5 studies [28,43,48,51,54] that included subjects in gingival/periodontal health status or with gingivitis [28,43,48,51,54], which was defined as mild or moderate in 3 studies [48,51,54].

Four studies [42,43,48,54] reported the absence of comorbidities that could affect the periodontal status and/or oral hygiene practices, while no study reported the presence of comorbidities.

One study [42] reported the minimum duration of fixed orthodontic treatment of 1 month; all other studies lacked data.

All included studies evaluated periodontal clinical parameters. In detail, GI was assessed (n = 23) in 12 studies [28,29,34,42,46,47,48,50,51,52,53,54]; PI (n = 26) in 13 studies [27,28,29,34,42,43,44,45,47,48,50,52,53]; GBI (n = 12) in 6 studies [27,28,45,48,52,54]; PPD has been reported (n = 8) in 4 studies [29,47,49,54]; BoP (n = 2) in 2 studies [29,46]; BI (n = 4) in 2 studies [27,47]; VPI (n = 4) in 2 studies [27,28]; OPI (n = 2) in 2 studies [42]; HI (n = 2) in 2 studies [29,48]. CPI (n = 1) [29], PBI (n = 1) [48], [27] MPI (n = 1) [27], and BBI [48] were recorded in 1 study. CAL was never registered since the investigated population was periodontally healthy as per the presently applied eligibility criteria.

No study recorded radiographic parameters, 2 studies recorded gingival crevicular parameters [28,50], and 3, other parameters [28,29,50].

### 3.3. Reported Evidence on Periodontal Outcomes in Orthodontic Patients with Fixed Appliances in Relation to Periodontal Self-Care Instructions, Prescriptions, and Motivational Methods

#### 3.3.1. Manual and Powered Toothbrushes in Periodontal Health Management of Orthodontic Patients with Fixed Appliances

Periodontal parameters were evaluated in 6 studies [42,43,45,46,49,54] in relation to the use of manual [42,43,49], orthodontic [45], or powered toothbrushes [46,54], individually or in combination, and with or without the addition of antimicrobial gels.

In all cases, at least one control group with manual or powered brushing was compared with the corresponding one. In three studies [43,46,54], the investigated manual [43], orthodontic [43], or powered [43,46,54] brushing system was specified in more detail, and one study [49] indicated the type of antimicrobial gel associated with manual brushing.

The duration of intervention use was detailed in 4 of these studies [42,45,46,49], with a minimum duration of 15 days [45] and a maximum duration of 23 months [49].

Follow-up was reported in 4 studies [45,46,49,54], with a minimum period of 15 days [45] and a maximum period of 12 months [54].

No significant improvements related to the use of manual toothbrushes were reported for GI [42], PI [42], OPI [42], and PPD [49] in 2 studies [42,49]. Instead, significant beneficial effects on PI were found at the 1-month follow-up in 1 study [43].

Similarly, significant improvements in PI related to orthodontic toothbrushes were revealed in another study [45], although not associated with beneficial effects on GBI [45].

Regarding the powered toothbrushes, a significant improvement was noted for PPD values [54] in the short term; for GBI [54] and GI [54] in the short and long term; but not for BoP [46], GI [46], and PPD [54] in the long term.

#### 3.3.2. Chlorhexidine-Containing Products in Periodontal Health Management of Orthodontic Patients with Fixed Appliances

The periodontal parameters were assessed in 3 studies in relation to the administration of chlorhexidine products [44,47,48]; as a mouthwash in 3 studies [44,47,48]; and in 1 study in the form of gels, toothpaste, or varnishes [47].

All 3 studies [23,24,26] provided information on the percentage of chlorhexidine administered.

The durations of chlorhexidine product use and follow-up to assess periodontal parameters were also reported in all 3 studies [44,47,48], with a minimum duration of 1 day [48] and a maximum of 8 months [44].

Chlorhexidine gel did not significantly affect PPD values [47].

Similarly, varnish did not exert significant beneficial effects on GI and PI [47], or as toothpaste on GI and BI [23], albeit positively influencing OPI [47].

#### 3.3.3. Other Organic Products in Periodontal Health Management of Orthodontic Patients with Fixed Appliances

Periodontal parameters were evaluated in subjects undergoing fixed orthodontic treatment and using organic products in 3 studies [27,28,29].

The organic product type or concentration was always reported.

The duration of the administration was recorded in 2 studies [27,28], with 30 min [28] being the minimum and 6 months being the maximum [27].

The timing of follow-up was reported in 2 studies [28,29], with the shortest being 30 min [28] and the longest being 8 weeks [29].

Two studies [27,29] evaluated the clinical periodontal parameters, specifically PPD [29], BoP [29], GI [29], CPI [29], PI [27,29], HI [29], GBI [27], BI [27], MGI [27], VPI [27], and MPI [27], but no evaluable measurements were reported.

One study [28] reported significant improvements in GBI and VPI in the group taking Matricaria chamomilla L, and PI and GI in the aloe vera group.

#### 3.3.4. Probiotics in Periodontal Health Management of Orthodontic Patients with Fixed Appliances

The periodontal parameters were evaluated in subjects taking probiotics during fixed orthodontic treatment in 2 studies [50,51].

Probiotics’ type and doses were clearly specified, and the duration of the intake ranged between 2 weeks [50,51] and 23.8 months [51]. The timing of follow-up was reported in only one study [50].

One study [50] evaluated PI and GI, but no evaluable measurements were reported; the other study [51] revealed no statistically significant improvements in GI.

#### 3.3.5. Motivational Methods in Periodontal Health Management of Orthodontic Patients with Fixed Appliances

Periodontal parameters were assessed in subjects undergoing fixed orthodontic treatment and approached through motivational methods by computer aids or other means for maintaining good oral hygiene in 3 studies [34,52,53].

Intervention modalities were reported in all cases. In 2 studies [34,52], the duration of intervention ranged from 6 weeks [34] to 12 months [34,52]. The timing of follow-up was reported in 2 studies [34,52], with the shortest being 1 month [52] and the longest 12 months [34,52].

Periodontal parameters that significantly benefited from motivational methods were GI [34] and PI [34] at the 3-month follow-up but not PI at the 1-month follow-up [34]. GI and PI were also assessed in 2 other studies [52,53], but no evaluable values were reported. GBI and PI were recorded in relation to smartphone apps and showed significant improvements after 6 months of use, but not before at a 3-month follow-up [52].

Table 2 summarizes the main findings from the studies included in this umbrella review concerning periodontal outcomes in relation to self-care instructions, prescriptions, and motivational methods.

## 4. Discussion

Considering that lifelong periodontal self-care education, motivation, and guidance are prerequisites for maintaining healthy periodontal conditions [35], and that biofilm control may be challenging in periodontally healthy orthodontic patients with fixed appliances [6,7,8], the present review aimed to characterize periodontal self-care instructions, prescriptions, and motivational methods; to evaluate and compare the associated periodontal outcomes; and to provide integrated evidence-based recommendations for periodontal self-care in periodontally healthy patients undergoing fixed orthodontic treatment.

A total of 17 studies [27,28,29,34,42,43,44,45,46,47,48,49,50,51,52,53,54] were included in the present umbrella review, with a total of 7547 periodontally healthy patients between the ages of 8 and 64 years undergoing fixed orthodontic treatment. The present study population reflects the sociodemographic characteristics of the current population of orthodontic patients [55]. Indeed, while orthodontic patients aged 19 years or older were rare in the 1960s, the number of adults undergoing orthodontic treatment increased exponentially by 2000. In 2006, older (≥40 years) adults comprised an estimated 4.2% of the orthodontic population, with 20% of patients over 60 years of age. Notably, considering the higher prevalence of periodontitis with increasing age [56], only periodontally healthy adult orthodontic patients fit the topic of the present study. Consistent with the present study’s sample, which has an M:F ratio of 1:1.5, a higher prevalence of female patients with fixed orthodontic appliances is generally found in every other age group [55]. None of the studies included in this umbrella review described limiting health impairments or comorbidities that might affect periodontal health status, but few reported their absence. It is well-known that some physical (e.g., disabling osteoarthritis, rheumatoid arthritis, and other musculoskeletal disorders [57]) and mental [58,59] disabilities can make it difficult to practice good oral hygiene.

Additionally, systematic conditions (e.g., neoplastic diseases) that may affect the periodontal supporting tissues independent of dental plaque biofilm-induced inflammation have been reported [60]. Given the above, the presence/absence of limiting health impairments and comorbidities that potentially affect periodontal health status and/or oral hygiene should be considered a confounder in this type of study and should be specified.

Most of the studies reviewed did not specify a fixed orthodontic treatment duration. However, this does not seem relevant because enamel demineralization and soft tissue inflammation may develop rapidly within the first few months of treatment, depending more on the individual’s susceptibility than on the treatment duration [61].

### 4.1. Periodontal Health Management of Orthodontic Patients with Fixed Appliances: Self-Care Instructions, Prescriptions, and Motivation Reinforcement

#### 4.1.1. Manual and Powered Toothbrushes

Brushing teeth as a daily routine is the most important healthy behavior to maintain oral and periodontal health [22]. However, the effectiveness of toothbrushing in removing biofilm depends on several factors, including the frequency and duration of daily toothbrushing [61], motivation, as well as knowledge, and manual dexterity [62]. Indeed, people without dental training rarely manage to clean more than 30–40% of their dental cervical area by manual toothbrushing [63,64], whereas dental professionals manage to clean more than 90% of their gingival margins [65].

Powered toothbrushes were introduced in the early 1960s as an alternative to manual methods [62]. They can be classified according to their mode of action (rotational oscillation; lateral oscillation; counter-oscillation; circular; ultrasonic; ionic), and their advertising slogans promise they provide a superior clean [66]. This assumption is supported by the systematic review by Yaacob et al. [67], which showed a slight, albeit significant, advantage of certain designs of electric toothbrushes over manual toothbrushes in reducing oral biofilm and preventing gingivitis. However, this study did not focus on orthodontic patients.

However, according to the findings from all the systematic reviews included, no significant differences between manual toothbrushes and electric toothbrushes in the efficacy in the mechanical control of bacterial plaque could be highlighted in patients undergoing fixed orthodontic treatment. ElShehaby et al. [42], comparing manual with powered toothbrushes, found slight and non-significant differences in GI, PI, and OPI at 4- and 8-week follow-ups. Accordingly, Kaklamanos et al. [46] found no difference between powered and manual toothbrushing in gingival inflammation (GI and BoP score). Conversely, Al Makhmari et al. [54] found an overall statistically significant advantage of powered over manual toothbrushes in terms of GI, GBI, and PD, but the authors acknowledged that more studies with low risk of bias, longer follow-up time, and broader samples are needed to provide solid evidence [54].

Pithon et al. [43] reported conflicting results comparing powered and convectional manual toothbrushes in orthodontic patients and concluded that brushing with a manual toothbrush twice daily for 1 to 3 min effectively reduced PI [43].

A study included in this systematic review analyzed orthodontic toothbrushes: special manual devices designed to provide adequate oral hygiene in orthodontic patients by using a V-shaped groove with shorter nylon bristles along the long axis of the toothbrush head to increase the contact area between the bristles of the toothbrush and the orthodontic appliance [25]. The orthodontic toothbrushes achieved more extensive plaque removal, although no differences in gingival bleeding were observed. This highlights the need for further clinical studies to obtain clinical recommendations [25].

#### 4.1.2. Chlorhexidine-Containing Products

Chlorhexidine (CHX) is a cationic compound capable of binding negatively charged bacterial cell walls and causing the rupture of bacterial cytoplasmic membranes, leading to cell death [68]. CHX is effective against both Gram-positive and Gram-negative bacteria, including aerobes and anaerobes [69].

The bactericidal spectrum and high substantivity in the oral cavity make CHX the gold standard for the chemical control of oral biofilm [26]. Accordingly, it is the most common antiseptic used for a limited period as an adjunct to mechanical therapy for periodontitis [70,71].

Regarding the efficacy of CHX in reducing plaque and gingival inflammation in orthodontic patients with fixed appliances, relevant evidence emerged from studies included in the present umbrella review. Karamani et al. [48] found significantly lower gingival inflammation and plaque accumulation in patients using CHX mouth rinses than in non-users. Hussain et al. [47] compared CHX-containing products (mouthwashes, toothpaste, gels, tooth varnish) with placebo or sodium fluoride products and found significant clinical improvements after administering CHX-containing mouthwashes, with a reduction in gingival inflammation (lower GI and BI score) and plaque accumulation (lower PI score). A dose–response relationship was noted, as 0.12% CHX mouth rinses had half the effect on GI as 0.20% CHX rinses. Periodontal probing depth (PPD) values were also significantly reduced by CHX mouth rinses [47].

In addition, a greater reduction in gingival index (GI) and bleeding index (BI) values was observed in the group using CHX-containing mouth rinses than in the group using fluoride-containing (sodium fluoride) mouth rinses. This result should not be surprising because fluoride ions are known to prevent tooth demineralization by inhibiting carbohydrate utilization by oral bacteria [72,73], although they do not alter the biofilm ecosystem [74]. Accordingly, the potential anti-plaque effect of some fluoride salts (especially stannous fluoride) may be due to the tin content [75].

The efficacy of CHX mouth rinses was also reported by Pithon et al. [44], who studied different types of mouth rinses containing organic molecules and fluorides (CHX, octedins, essential oil, cetylpyridium, sodium fluoride, and amine fluoride/stannous fluoride) and found them effective in reducing biofilm accumulation (low PI) in orthodontic patients. Finally, Fatima et al. [49] found a significant improvement in gingivitis but not PPD after applying chlorhexidine or other antimicrobial gels.

Conversely, no clinically relevant benefits were found for CHX-containing toothpastes, gels, or varnishes [47]. The authors believe such findings may be ascribable to the greater ease and related treatment compliance of mouthwash compared to gels and other formulations, especially in patients with fixed orthodontic appliances [47]. Karamani et al. [48] also found a significantly lower gingival inflammation and plaque accumulation in patients with CHX mouthwashes than in control groups [48]. The efficacy of CHX mouthwashes was also reported by Pithon et al. who investigated different types of organic molecules- and fluorides-containing mouthwashes (CHX, octedine, essential oil, Cetylpyridium, sodium fluoride, and amine fluoride/stannous fluoride) and found them effective in the reduction of the accumulation of plaque (lower PI) in orthodontic patients. Lastly, Fatima et al. [38] noted a significant improvement in gingivitis by using chlorhexidine or other antimicrobial gels, but no significant differences in the probing depth between antimicrobial agents and the control group.

However, despite the beneficial effects of CHX-containing products on achieving and maintaining periodontal health, the case-specific benefit/risk ratio should be accurately assessed before CHX administration [76]. Indeed, from a clinical point of view, the potential adverse effects of CHX, such as dry mouth, change in taste, discoloration of the teeth, and hypersensitivity reactions, should be considered [76].

#### 4.1.3. Other Organic Products

In addition to CHX, other organic molecules have been shown to exert antimicrobial effects against oral species, including other biguanides (octenidine, alexidine), quaternary ammonium salts (cetylpryridinium and benzalkonium chloride), and pyrimidine derivatives (hexidine) [77]. Periodontal outcomes following the administration of these antimicrobials were also evaluated in the study mentioned above by Pithon et al. [44].

More recently, the potential beneficial effects of natural products, such as herbs and plant extracts, on oral mucosa and gingiva have been investigated [78]. In detail, herbal mouthwashes containing natural compounds with anti-inflammatory and antimicrobial activity, such as the essential oil of *Matricaria chamomilla* L. [78], *Sanguinaria canadensis, Eucalyptus globulus*, *Salvadora persica*, *Azadirichta indica* [79], *Zingiber officinale* [80], *Prunus mume* [81], and *Aloe vera* [82] have been tested as methods for biofilm control. According to Panagiotou et al. [27], some herbal mouthwashes (notably *Matricaria chamomilla* L., *Zingiber officinale*, and *Prunus mume*) appeared to be effective in reducing oral biofilm accumulation and/or gingival inflammation in patients with fixed orthodontic appliances. Papadopoulou et al. [28] also found promising results for an aloe vera mouth rinse, honey ingestion, and chamomile mouth rinse in reducing biofilm and gingival bleeding.

However, when comparing the efficacy of herbal mouthwashes with CHX-based ones in patients undergoing fixed orthodontic treatment, Kommuri et al. [29] reported conflicting results, as few studies were found with a high risk of bias.

#### 4.1.4. Probiotics

According to the definition of the WHO/FAO [30], probiotics are living microorganisms that provide health benefits to the host when administered in certain amounts. Among the beneficial effects of probiotics, there is some evidence of their role in disrupting gingival biofilm and modulating the host immune response. However, the exact mechanism of action is still unknown [31,32]. Since biofilm has been implicated in the pathogenesis of caries and periodontal disease, and the latter is also associated with the host response, it is suggested that probiotics may be useful in the prevention and treatment of these diseases [83,84]. However, the evidence for probiotics’ clinical efficacy in the prevention of caries and periodontal health management is still inconclusive [33,85,86].

Probiotic administration has also been suggested to be effective in improving or maintaining oral health in patients treated with fixed orthodontic appliances, as they are at a greater risk for caries and gingivitis development due to biofilm accumulation favored by the appliances [87,88].

However, the two relevant systematic reviews we currently considered showed contradictory results. According to Hadj-Hamou et al. [51], there is moderate evidence that probiotics do not affect gingival inflammation in these patients. Instead, Pietri et al. [50] found that the administration of probiotics decreased the number of pathogenic bacteria in oral biofilm and/or saliva, facilitating the maintenance of oral health. They also reported a possible mild effect of probiotics in reducing biofilm accumulation and gingivitis. However, the studies included in their systematic review had a moderate risk of bias due to heterogeneity in the methodology, and the recorded outcomes remain questionable because the preliminary sample size calculation was hardly performed [50].

Therefore, the results of both studies should be interpreted with caution. From a clinical perspective, further well-designed RCTs with a longer follow-up period are needed to evaluate the role of probiotic administration in maintaining oral health in patients undergoing fixed orthodontic therapy.

#### 4.1.5. Motivational Methods

Dental caries, biofilm accumulation, and gingivitis are primarily due to unhealthy self-care behaviors. Patients are usually instructed in oral hygiene relevance and related procedures by dentists. However, conventional oral health education, which focuses on disseminating information and giving instructions, often does not lead to a change in misbehavior [89].

According to Huang et al. [34], patient motivation may be critical in maintaining a behavioral change. Indeed, when investigating different motivational methods, they found a statistically significant improvement in plaque accumulation (lower PI score) and gingival inflammation (lower GI score) in subjects who underwent motivational interventions compared to control subjects.

Several methods could enhance patient motivation, and the most promising tools seemed to be reminders sent to patients via mobile health applications [90,91,92,93] or text messages [94,95,96].

In orthodontic patients, Sharif et al. [52] concluded that apps and mobile phone-based reminders could be effective behavior-change techniques to improve compliance with oral hygiene instructions during treatment.

Data collected by Migliorati et al. [53] showed that regular patient motivation sessions with one-to-one instruction by a hygienist also help maintain good oral hygiene in patients undergoing fixed orthodontic treatment.

### 4.2. Periodontal Health Management of Orthodontic Patients with Fixed Appliances: Self-Care Instructions-Related Outcomes and Evidence-Based Recommendations

#### 4.2.1. Biofilm Control

Electrically powered and manual toothbrushes did not show significant differences in the plaque index (PI) [42,43], so they can be considered equally effective for mechanical biofilm control in orthodontic patients. Instead, orthodontic brushes could provide better control of biofilm accumulation [45].

CHX mouthwashes, but not other CHX-containing products (gels, varnishes, pastes), may be better used to control plaque accumulation in addition to tooth brushing, but only for a limited period [44,47,48]. Considering the side effects of CHX, other organic products or herbal mouthwashes may be recommended, as they significantly reduced the PI score [27,28,44], and their effectiveness seemed comparable to CHX ones [29].

Probiotics did not show significant results in terms of PI score improvements [50], while motivational methods have proved to be a simple and effective means of maintaining good biofilm control [34,52,53].

#### 4.2.2. Gingival Inflammation Reversal

Conflicting findings related to the type of toothbrush used and gingival inflammatory parameters. Indeed, some results suggest that the use of electric or manual (including orthodontic) toothbrushes has no direct beneficial effect on inflammatory periodontal parameters—gingival bleeding index (GBI) [42,45], GI, or bleeding on probing (BoP) scores [46]—and PPD [49]. Other findings, instead, revealed an improvement in these indices, particularly GI, GBI scores, and PPD scores, in the short term [54] with powered toothbrushing.

CHX-based [47,48] and herbal-based mouthwashes [27,28,29] are associated with lower GBI, GI scores, and PPD values than control groups. Therefore, the use of these antimicrobial agents to maintain oral gingival health in patients with fixed appliances should be considered.

In contrast, no beneficial effects on GI currently support probiotics administration [50,51].

Active reminders to motivate patients on a regular basis represent an effective intervention to limit gingivitis in orthodontic patients [34,52,53].

#### 4.2.3. Evidence-Based Periodontal Self-Care Recommendations for Periodontally Healthy Orthodontic Patients with Fixed Appliances

Patients should be encouraged to brush their teeth at least twice daily with a manual toothbrush or an electric toothbrush for 1 to 3 min, depending on their preference [42,43,45,46,49].

If orthodontic patients with fixed appliances are unable to control biofilm accumulation with conventional toothbrushes, the use of specifically designed devices (orthodontic toothbrushes) may be prescribed [45].

When patients undergoing fixed orthodontic treatment are unable to maintain good oral hygiene by toothbrushing alone, additional chemical biofilm control should be considered [27,28,29,43,47,48].

A risk–benefit assessment should always be performed before prescribing chlorhexidine, because, despite being the best-studied and most effective oral antiseptic [97], its use may be associated with adverse reactions [98].

If the patient’s periodontal conditions are considered at high risk for disease development, and no history of hypersensitivity reactions to CHX is reported, chlorhexidine-containing mouthwashes should be administered in the absence of hypersensitivity reactions. Concentrations of 0.12% to 0.20% should be prescribed, as the efficacy of lower concentrations remains uncertain [99], and higher ones unnecessarily increase side effects [98]. The CHX regimen was described in a recent Cochrane systematic review [76].

If the patient’s periodontal conditions are considered at low/moderate risk of disease development or a history of type I and type IV hypersensitivity reactions associated with oral use of CHX is reported, other organic molecules (octedin, cetylpyridinium chloride, sodium fluoride, amine fluoride/tin fluoride, essential oil)-containing mouthwashes should be preferred [27,28,29,43]. These mouthwashes should be administered according to the manufacturer’s instructions.

Routine oral hygiene instructions should be repeated during treatment and reinforced by motivational methods. Cell phones are essential tools for improving adherence to oral hygiene instructions, especially in children and adolescents [34,52,53].

**Figure 3:**
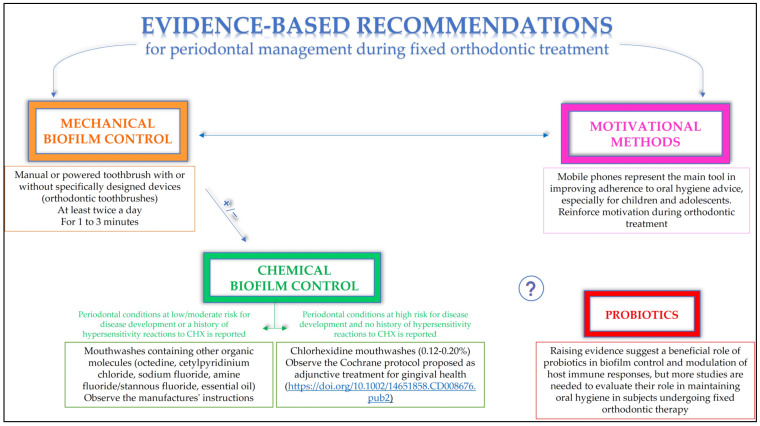
Evidence-based recommendations for periodontal management in periodontally healthy orthodontic patients with fixed appliances [76].

Figure 3 summarizes the evidence-based recommendations for periodontal management in periodontally healthy orthodontic patients with fixed appliances.

The heterogeneity of the extracted data, especially regarding the timing of the follow-up, has precluded the possibility of performing a meta-analysis, which is the study’s main limitation. In addition, heterogeneous data, particularly regarding the timing of follow-up, and missing data on administration regimens and intervention duration, precluded the possibility of conducting a meta-analysis, which is the study’s main limitation.

However, the present umbrella review may be the first to comprehensively characterize periodontal self-care instructions, prescriptions, and motivational methods, attempting to provide recommendations for periodontal self-care instructions and methods in periodontally healthy orthodontic patients with fixed appliances.

Further studies should highlight the most effective self-care instructions and methods, individually and in combination, to define standardized periodontal health management protocols for orthodontic patients with fixed appliances.

## 5. Conclusions

The present umbrella review included 17 systematic reviews investigating the periodontal parameters in healthy subjects in fixed orthodontics in relation to manual, orthodontic, or powered toothbrushes; CHX-containing or other organic products; probiotics; and motivational methods.

Powered and manual toothbrushes showed no significant differences in the PI score increase. However, some evidence revealed a significant improvement in GI, GBI, and PPD in the short term offered by powered toothbrushes.

CHX mouthwashes, but no other CHX-containing products (gels, varnishes, pastes), have been proposed to better control biofilm accumulation and gingival inflammation in addition to toothbrushing, but only for a limited period.

The effectiveness of other organic products due to their antimicrobial properties was reported for aloe vera and chamomile and seemed comparable to CHX without its side effects in the long term, particularly for herbal-based mouthwashes.

Motivational methods also showed beneficial effects on biofilm accumulation and gingival inflammation, while no evidence has been found on the effectiveness of probiotics.

Therefore, at the current state of knowledge, the gold standard for biofilm control and gingival inflammation reduction in subjects with fixed orthodontic treatment may be the combination of manual, orthodontic, or powered brushing; motivational aids; and organic products, or the short-term use of CHX mouthwashes.

Future research should determine standardized periodontal self-care protocols for optimal periodontal health management in orthodontic patients with fixed appliances.

## Figures and Tables

**Table 1:** Data collected from the studies included in the present umbrella review: general information: first author, year, journal of publication, reference number, meta-analysis, funding, quality; methods: characteristics of the study (design and number), participants (sample size, age, gender, periodontal status, comorbidities potentially affecting periodontal status, and/or oral hygiene practice, fixed orthodontic treatment duration), intervention (type, characteristics, duration, and follow-up), and comparison; periodontal outcomes statistically significant: clinical and radiographic periodontal parameters and gingival crevicular inflammatory mediators; conclusion(s).

Included Studies	Methods	Periodontal Outcomes Statistically Significant (*p* < 0.05)	Conclusion(s)
Authors, Year […] Journal Meta-analysis Funding Quality	Studies (design and number) Population Sample size: (n.) Mean age: (y.o.) Male/Female ratio: (M/F) Periodontal status Comorbidities potentially affecting the periodontal status and/or oral hygiene practice Fixed orthodontic treatment duration: (mo.) Intervention Type Characteristics Duration Follow-up Comparison Any	Clinical Clinical Attachment Level (CAL) Periodontal Probing Depth (PPD) Bleeding on Probing (BoP) Gingival Bleeding Index (GBI) Bleeding Index (BI) Gingival Index (GI) Modified Gingival Index (MGI) Plaque Index (PI) Visible Plaque Index (VPI) Modified Plaque Index (MPI) Orthodontic Plaque Index (OPI) Community Periodontal Index (CPI) Papilla Bleeding Index (PBI) Bonded Bracket Index (BBI) Hyperplastic Index (HI) Radiographic Any Gingival crevicular Any Others Any	Synthesis of findings from the systematic review presently included.
ElShehaby M., 2020 [42] Am J Orthod Dentofacial Orthop. Meta-analysis No funding Low quality	Studies n = 7 RCT (n = 7) Population Sample size: n = 423 Mean age: 10—up to 20 y.o. Male/Female ratio: 172M/251F Periodontal status: NDF Comorbidities potentially affecting the periodontal status and/or oral hygiene practice: none Fixed orthodontic treatment duration: at least 1 mo. Intervention Manual toothbrush Characteristics: NDF Duration: mean: 8.86 w; from 4 to 20 w Follow-up: NDF Comparison Powered toothbrush	Clinical At 4 and 8 w follow-up GI: NSS PI: NSS OPI: NSS	There were slight differences in GI, PI, and OPI at 4 and 8 w follow-up favoring powered brushing, but this difference was not statistically significant.
Fatima F., 2020 [49] Int Orthod. Meta-analysis No funding Critically low quality	Studies n = 7 RCT (n = 4) Quasi-experimental trials (n = 3) Population Sample size: n = 477 Mean age: 10.4 to 20 y.o. Male/Female ratio: 125M/127F/225NDF Periodontal status: NDF Comorbidities potentially affecting the periodontal status and/or oral hygiene practice: NDF Fixed orthodontic treatment duration: NDF Intervention Manual toothbrush + antimicrobial gels Characteristics: antioxidant-essential oil gel/amine fluoride gel/0.4% stannous fluoride gel/0.2% or 2% CHX gel/0.3% triclosan-containing dental gel Duration: 1-time application—23 mo. Follow-up: from 2 w to 12 w Comparison Only manual toothbrush Manual toothbrush + placebo Manual toothbrush + antimicrobial gels at different concentrations	Clinical At 2 w follow-up PPD: NNS At 4 w follow-up PPD: NNS	In the 2 and 4 w follow-ups, no significant differences were observed in the antimicrobial group compared to the control group concerning PPD.
Pithon M.M., 2017 [43] Biosci J Meta-analysis No funding Critically low quality	Studies n = 23 RCT (n = 23) Population Sample size: n = 1022 Mean age: 10 to 53 y.o. Male/Female ratio: 295M/408F/319 NDF Periodontal status: healthy/gingivitis Comorbidities potentially affecting periodontal status and/or oral hygiene practice: none Fixed orthodontic treatment duration: NDF Intervention Manual/orthodontic/Siwak/Siwak and orthodontic/interdental and orthodontic/ultrasonic brush/manual brush and mouthwash/oral irrigation/oral irrigation and dental floss/oral irrigation appliance and automatic brush or manual brush/electric brush with orthodontic head/dental floss Characteristics: Manual brush: Oral-B Model 30 or 35/Oral-B SensitiveOral-B Advantage/Elmex 29/Elmex^®^ interX/Gum Super Tip/Gum 311 Orthodontic brush: Oral-B Orthodontic/Lactona Orthodontic/Oral-B 15 Electric brush: Braun Oral-B 3D/Oral-B Cross Action/Plaque Remover/Braun with orthodontic head OD5-1 or HP550 with HO5924 head/Interplak/Rota-dent/Plaque remover EB5 or OD5/Philips-Jordan Interdental brush: Oral-B/TePe^®^/Elmex 1283 Compact Tuft with a long straight handle/Elmex^®^ interdental brush No. 6 with a short, curved handle/WaterPik Flosser Automatic brush: Plaque Control 2000 Ultrasonic brush: Ultrasonx Ultima Toothbrush^®^ Dental floss: Oral-B/Elmex^®^ multi-floss/WaterPik^®^ Sonic Speed or Flosser Oral irrigation appliance: WaterPik^®^ Sonic Speed sonic/Sonic Speed Mouthwash: Kin with 0.12% CHX and 0% alcohol Duration: NDF Follow-up: NDF Comparison Different mechanical methods from that of the intervention	Clinical Manual brush At 1 mo. Follow-up PI: (MD: −1.01; 95% CI; −1.23 to −0.79) *p* < 0.001	In orthodontic patients, the conventional manual brush was effective for mechanical control of bacterial plaque.
Marçal F.F., 2022 [45] Int J Dent Hyg. Meta-analysis No funding Critically low quality	Studies n = 6 RCT (n = 3) Non-RCT (n = 3) Population Sample size: n = 243 Mean age: 8 to 40 y.o. Male/Female ratio: 98M/125F/20NDF Periodontal status: NDF Comorbidities potentially affecting the periodontal status and/or oral hygiene practice: NDF Fixed orthodontic treatment duration: NDF Intervention Orthodontic toothbrush Characteristics: NDF Duration: from 15 d to 6 mo. Follow-up: from 15 d to 6 mo. Comparison Conventional toothbrush	Clinical GBI: NSS PI: (MD: −1.72; 95% CI; −0.83 to −2.61; 82% l2) *p* = 0.0001	GBI was not modified by an orthodontic design toothbrush. The use of an orthodontic toothbrush greatly improved PI instead of the use of a conventional toothbrush.
Al Makhmari S.A., 2017 [54] Am J Orthod Dentofacial Orthop. Meta-analysis No funding Low quality	Studies n = 9 RCT (n = 9) Population Sample size: n = 434 Mean age: 11.4 to 19.25 y.o. Male/Female ratio: 145M/168F/121NDF Periodontal status: healthy/mild or moderate gingivitis Comorbidities potentially affecting periodontal status and/or oral hygiene practice: none Fixed orthodontic treatment duration: NDF Intervention Powered toothbrush Characteristics: side-toside/counter-oscillation/rotation-oscillation/circular-acting toothbrush/ultrasonic/ionic-toothbrush/unknown action toothbrush/toothbrush with other mechanism of action Duration: mean 4 mo. (from 3 to 12 mo.) Follow-up: 3–12 mo. Comparison Manual toothbrush	Clinical Short-term powered toothbrushes PPD: (WMD: −0.760; 95% CI; −1.029 to −0.491; n = 24) *p* = 0.000 GBI: (SMD: −0.637; 95% CI; −1.092 to −0.183; 95% Pi; −2.106 to −0.832; n = 342; l2 = 76%) *p* = 0.06 GI: (WMD: −0.079; 95% CI; −0.146 to −0.012; 95% Pi; −0.300 to 0.142; n = 374; l2 = 83%) *p* = 0.021 Long-term powered toothbrushes PPD: NSS GBI: (WMD: −1.630; 95% CI; −3.206 to −0.054; n = 40) *p* = 0.043 GI: (WMD: −0.220; 95% CI; −0.424 to −0.016; n = 40) *p* = 0.035	In the short term, powered toothbrushes provided an overall statistically significant benefit compared with manual toothbrushes with regard to the GI and GBI. In the long term, only 1 study showed a statistically significant benefit with regard to both the GI and GBI. With regard to probing pocket depth, there was a statistically significant benefit of powered over manual toothbrushes in the short term but not in the long term.
Kaklamanos E.G., 2008 [46] Am J Orthod Dentofacial Orthop Meta-analysis No funding Critically low quality	Studies n = 5 RCT (n = 5) Population Sample size: n = 304 Mean age: older than 11 y.o. Male/Female ratio: 77M/94F/133NDF Periodontal status: NDF Comorbidities potentially affecting the periodontal status and/or oral hygiene practice: NDF Fixed orthodontic treatment duration: NDF Intervention Powered toothbrush Characteristics: Rotation oscillation action toothbrush/side-to-side action toothbrush/ionic toothbrush/toothbrush with bristles pulsating at 6000 strokes per minute Duration: 60 d Follow-up: 60 d Comparison Manual toothbrush	Clinical BoP: NSS GI: NSS	No statistically significant difference between powered and manual toothbrushing for GI or BoP was noted.
Hussain U., 2022 [47] Eur J Orthod. Meta-analysis No funding Low quality	Studies n = 20 RCT (n = 20) Population Sample size: n = 1001 Mean age: 14–9 to 15.4 y.o. Male/Female ratio: 103M/279F/619NDF Periodontal status: NDF Comorbidities potentially affecting the periodontal status and/or oral hygiene practice: NDF Fixed orthodontic treatment duration: NDF Intervention CHX mouthwash/gel/toothpaste/varnishes Characteristics: 0.06%/0.12%/0.2%/0.5%/0.75%/0.95%/1%/2% of CHX Duration: from 1 mo. to 6 mo. Follow-up: 1–3–6 mo. Comparison No intervention Placebo Sodium fluoride products (mouthwash, gel, toothpaste, varnishes)	Clinical CHX mouthwash vs. placebo At 1 mo. follow-up GI: (MD: −0.67; 95% CI; −0.92 to −0.42; n = 3) *p* < 0.001 PI: (MD: −0.71; 95% CI; −0.90 to −0.52; n = 3) *p* < 0.001 At 3 mo. follow-up PPD: (MD: −0.60; 95% CI; −1.06 to −0.14; n = 2) *p* < 0.01 BI: (MD: −1.61; 95% CI; −2.99 to −0.22; n = 3) *p* < 0.02 GI: (MD: −0.68; 95% CI; −0.97 to −0.38; n = 9) *p* < 0.001 PI: (MD: −0.65; 95% CI; −0.86 to −0.43; n = 9) *p* < 0.001 At 6 mo. follow-up BI: (MD: −0.90; 95% CI; −1.39 to −0.40; n = 2) *p* < 0.001 GI: (MD: −0.44; 95% CI; −0.86 to −0.02; n = 2) *p* < 0.04 PI: NSS CHX gel vs. placebo At 1 mo. follow-up PPD: NSS At 3 mo. follow-up PPD: NSS GI: NSS PI: NSS CHX varnish vs. placebo At 3 mo. follow-up GI: NSS PI: NSS At 6 mo. follow-up GI: NNS PI: NSS CHX toothpaste vs. sodium fluoride mouthwash At 3 mo. follow-up BI: NSS GI: NSS OPI: (MD: −5.24; 95% CI; −10.46 to −0.02; n = 2) *p* < 0.04	There were clinically relevant benefits from using CHX-containing mouthwashes on PPD, GI, PI, and GBI for the observation periods of 0–1 mo. or 1–3 mo., but not after 3–6 mo. No clinically relevant benefits were found for CHX-containing toothpaste, gel, or varnish.
Karamani I., 2022 [48] Oral Health Prev Dent. Meta-analysis No funding Low quality	Studies n = 14 RCT (n =14) Population Sample size: n = 602 Mean age: 11 to 35 y.o. Male/Female ratio: 200M/357F/45NDF Periodontal status: healthy/mild or moderate gingivitis Comorbidities potentially affecting the periodontal status and/or oral hygiene practice: none Fixed orthodontic treatment duration: NDF Intervention CHX mouthwash Characteristics: 0.12%/0.2/ 0.06%/N/D CHX Duration: from 1 d to 3 mo. Follow-up: from 1 min to 5 mo. Comparison Placebo/sterile isotonic saline/aloe vera/chlorine dioxide/MTC/isotonic saline with sodium chloride/propolis/probiotic/herbal antiseptic/Zingiber officinale essential oil/neem/CHX digluconate 0.06% and sodium fluoride 0.05%/CHX anti discoloration system -mouthwash	Clinical GBI: N/D CHX vs. propolis/probiotics/herbs at 3 and 4 w: GI: NSS PI: N/D PBI: N/D BBI: N/D HI: N/D	Statistically significant differences were revealed concerning GBI, GI, PI, PBI, BBI, HI, and PPD between the CHX group and control groups, especially in the first week. CHX reduced plaque accumulation and gingival inflammation more effectively than the placebo solution.
Pithon M.M., 2015 [44] J Dent. No meta-analysis No funding Critically low quality	Studies n = 15 RCT (n = 14) CCT (n = 1) Population Sample size: n = 638 Mean age: 11 to 33 y.o. Male/Female ratio: 158M/266F/ 214NDF Periodontal status: NDF Comorbidities potentially affecting periodontal status and/or oral hygiene practice: NDF Fixed orthodontic treatment duration: NDF Intervention Powered toothbrush CHX/Cetylpyridinium/ amine fluoride stannous fluoride/Octedine dihydrochloride/Polyvinylpyrrolidone-iodine/sodium fluoride/ essential oil-based mouthwash Sanguinaria-containing toothpaste Characteristics: CHX 0.2% CHX gluconate 0.12% Polyvinylpyrrolidone-iodine 7.5% Sodium fluoride 0.2% Amine fluoride stannous fluoride 250 ppm of F, pH 4.0 5% umbuzeiro fruti extract Duration: from 2 w to 8 mo. Follow-up: from 2 w to 8 mo. Comparison Placebo mouthwash No intervention	Clinical PI: N/D	The use of mouthwashes based on chlorhexidine, octenidine, essential oil, Cetylpyridinium, sodium fluoride, and amine fluoride/stannous fluoride was shown to be effective in reducing PI.
Papadopoulou, 2021 [28] Clin Exp Dent Res. No meta-analysis No funding Critically low quality	Studies n = 3 RCT (n = 3) Population Sample size: n = 135 Mean age: 12 to 40 y.o. Male/Female ratio: 44M/91F Periodontal status: healthy/gingivitis Comorbidities potentially affecting the periodontal status and/or oral hygiene practice: NDF Fixed orthodontic treatment duration: NDF Intervention Organic products Characteristics: MTC mouthwash Aloe vera mouth rinse Chew and ingest pure undiluted honey Duration: from 30 min to 15 d Follow-up: from 30 min to 15 d Comparison Placebo/sucrose/sorbitol/chlorine dioxide -mouthwash	Clinical MTC vs. placebo Placebo group GBI: +23.1% VPI: +10.2% MTC group GBI: −29.9% VPI: −25.6% CHX group GBI: −32.0% PI: −31.39 ± 16.58 GI: −16.30 ± 9.98 VPI: −39.9% Aloe vera group PI: −20.38 ± 16.74 GI: 9.88 ± 8.77 Chlorine dioxide group: PI: −30.29 ± 18.30 GI: −12.22 ± 9.30 Gingival crevicular Bacterial counts: N/D Others pH: N/D	MTC reduced PI and GBI patients with gingivitis. Chlorine dioxide can be a suitable alternative for CHX. Aloe vera was not equally effective. Bacterial counts were significantly reduced in the honey group compared to the other groups and inhibited bacterial growth significantly compared to inhibition observed with antibiotics. Honey topical application can modify the pH, reduce bacterial counts, and inhibit bacterial growth. The pH of the sorbitol group did not change.
Kommuri K., 2022 [29] Int J Dent Hyg. Meta-analysis No funding Critically low quality	Studies n = 8 RCT (n = 8) Population Sample size: n = 425 Mean age: 13 to 26 y.o. Male/Female ratio: 132M/171F/122NDF Periodontal status: NDF Comorbidities potentially affecting the periodontal status and/or oral hygiene practice: NDF Fixed orthodontic treatment duration: NDF Intervention Organic products Characteristics: Herbal-based mouthwash Duration: NDF Follow-up: from 3 d to 8 w Comparison CHX-based mouthwash	Clinical PPD: N/D BoP: N/D GI: N/D PI: N/D CPI: N/D HI: N/D Others CFU of oral bacteria: N/D	Two studies reported that oral hygiene maintenance properties of CHX-based mouthwashes were superior in reducing S. mutans count compared to organic products. One study showed that CHX-based mouthwashes improved PI and PPD parameters. Four studies showed that CHX was as effective as herbal-based mouthwashes.
Panagiotou A., 2021 [27] Int J Environ Res Public Health No meta-analysis No funding Critically low quality	Studies n = 6 RCT (n = 3) Non-RCT (n = 3) Population Sample size: n = 255 Mean age: 10 to 64 y.o. Male/Female ratio: 66M/110F/79NDF Periodontal status: NDF Comorbidities potentially affecting the periodontal status and/or oral hygiene practice: NDF Fixed orthodontic treatment duration: NDF Intervention Essential oil Characteristics: Mouthwash of Listerine^®^/Listerine Fructus mume 2.5%/ MTC 1%/Zingiber Officinale 0.5% Duration: from 1 w to 6 mo. Follow-up: NDF Comparison Mouthwashes that did not contain essential-oils (CHX, povidone-iodine, placebo, distilled water) No mouthwash	Clinical GBI: N/D BI: N/D MGI: N/D PI: N/D VPI: N/D MPI: N/D	Listerine^®^ was effective in decreasing PI and GBI. Fructus mume was effective in decreasing GBI. Zingiber officinale, MTC, and CHX effectively decreased GBI and oral biofilm accumulation. MTC and CHX were comparable for anti-inflammatory efficacy.
Pietri F.K., 2020 [50] Probiotics and Antimicrobial Proteins No meta-analysis No funding Critically low quality	Studies n = 9 RCT (n = 9) Population Sample size: n = 391 Mean age: 8 to 35 y.o. Male/Female ratio: 88M/166F/137NDF Periodontal status: NDF Comorbidities potentially affecting the periodontal status and/or oral hygiene practice: NDF Fixed orthodontic treatment duration: NDF Intervention Probiotics Characteristics: Mouthwash/lozenges/yogurt/curd/kefir/toothpaste with probiotic bacteria (Streptococcus salivarius M18 or K12; Lactobacillus paracasei/plantarum/acidophilus/reuteri) Duration: from 2 w to 17 ± 6.8 mo. Follow-up: from 2w to 17 ± 6.8 mo. Comparison No treatment Fluoridated mouthwash CHX mouthwash Placebo	Clinical GI: N/D PI: N/D Others Subgingival levels of Porphyromonas gingivalis: N/D Salivary streptococcal colony count: N/D Streptococcus mutans and Lactobacillus scores in plaque and saliva: N7D Halitosis: N/D	Seven studies showed that probiotics reduced the counts of oral pathogenic bacteria in the oral biofilm and/or saliva. One study reported that probiotics reduced halitosis. One study found that PT reduced PI and GI, while another study reported no significant influence on PI and GI.
Hadj-Hamou R., 2020 [51] BMC Oral Health No meta-analysis No funding Low quality	Studies n = 4 RCT (n = 4) Population Sample size: n = 237 Mean age: 10 to 30 y.o. Male/Female ratio: 77M/130F/30NDF Periodontal status: healthy/mild or moderate gingivitis Comorbidities potentially affecting the periodontal status and/or oral hygiene practice: NDF Fixed orthodontic treatment duration: NDF Intervention Probiotics Characteristics: Lozenges with Streptococcus salivarius M18 only or K12; Lactobacillus paracasei/plantarum/acidophilus/reuteri Drink with Lactobacillus casei strain Shirota Duration: from 2 w to 23.8 mo. Follow-up: NDF Comparison Placebo No intervention	Clinical GI: NSS	No statistically significant benefit was found regarding GI.
Huang J., 2018 [34] Medicine (Baltimore) Meta-analysis No funding Critically low quality	Studies n = 12 RCT (n = 10) Quasi-random (n = 1) CCT (n = 1) Population Sample size: n = 830 Mean age: 10 to 31 y.o. Male/Female ratio: NDF Periodontal status: NDF Comorbidities potentially affecting the periodontal status and/or oral hygiene practice: NDF Fixed orthodontic treatment duration: NDF Intervention Motivational methods and reminders Characteristics: Text message/repeated OHI/WhatsApp chat room-based competition and shared 2 self-photographs monthly leaflets/one-to-one instruction with a hygienist/specially made videotape/instruction-plus-persuasion Duration: from 6 w to 12 mo. Follow-up: from 6 w to 12 mo. Comparison No OHI Only OHI at baseline Written OHI	Clinical At 1 mo. follow-up GI: (MD: −017; Cl 95%; −0.23 to −0.11) *p* < 0.05 PI: NSS At 3 mo. follow-up GI: (MD: −0.20; CI 95%; −0.33 to −0.06) *p* < 0.05 PI: (MD: −0.23; Cl 95%; −0.39 to −0.06) *p* < 0.05 At 6 mo. follow-up GI: (MD: −0.30; Cl 95%; −0.36 to −0.23) *p* < 0.05 PI: (MD: −0.19; Cl 95%; −0.35 to −0.03) *p* < 0.05	Motivational methods had significant advantages regarding PI in the experimental group over the control group at 1, 3, and 6 mo. GI was significantly better controlled in the study group at 3–6 mo.
Sharif M.O., 2019 [52] Br Dent J No meta-analysis Royal College of Surgeons of England Faculty of Dental Surgery Critically low quality	Studies n = 2 RCT (n = 1) Non-RCT (n = 1) Population Sample size: n = 130 Mean age: 14.5 y.o. Male/Female ratio: 55M/75F Periodontal status: NDF Comorbidities potentially affecting the periodontal status and/or oral hygiene practice: NDF Fixed orthodontic treatment duration: NDF Intervention Any interventions delivered by mobile phones Characteristics: Text messages/smartphone video tutorials/mobile phone app (Brush Game) Duration: from 3 mo. to 12 mo. Follow-up: 1–3–6–9–12 mo. Comparison Any interventions delivered not using mobile phones (audio-visual presentation on how to brush correctly/standardized oral hygiene instructions to oral hygiene)	Clinical Text messages group GI: N/D PI: N/D Mobile phone app and smartphone video tutorials group At 3 mo. GBI: NSS PI: NSS At 6 mo. GBI: *p* < 0.01 PI: *p* < 0.01 At 9 mo. GBI: *p* < 0.05 PI: *p* < 0.01 At 12 mo. GBI: *p* < 0.05 PI: *p* < 0.01	PI was statistically significantly lower in the intervention group at the final follow-up. Mobile phone apps and smartphone video tutorials effectively reduced GBI at 6, 9, and 12 mo., but not at 3 mo.
Migliorati M., 2015 [53] Eur J Orthod. No meta-analysis No funding Critically low quality	Studies n = 10 RCT (n = 8) CCT (n = 1) before/after study (n = 1) Population Sample size: NDF Mean age: NDF Male/Female ratio: NDF Periodontal status: NDF Comorbidities potentially affecting the periodontal status and/or oral hygiene practice: NDF Fixed orthodontic treatment duration: at least 12 mo. Intervention Motivational methods and professional hygiene and prophylaxis Characteristics: Oral hygienist intervention Prophylaxis regime Communication techniques (written, visual, verbal) Duration: NDF Follow-up: NDF Comparison Usual care No intervention	Clinical GI: N/D PI: N/D	Regular patient motivational sessions and mechanical tooth cleaning by a professional dental hygienist helped maintain good oral hygiene during fixed orthodontics.

Abbreviations: randomized clinical trial, “RCT”; controlled clinical trial, “CCT”; male, “M”; female, “F”; years old, “y.o.”; number, “n”; month(s), “mo.”; week(s), “w”; day(s), “d”; minute(s), “min”; parts per million, “ppm”; not defined, “N/D”; no data found, “NDF”; not statistically significant, “NNS”; Chlorhexidine, “CHX”; Matricaria chamomilla L, “MTC”; oral hygiene instruction, “OHI”; clinical attachment loss, “CAL”; periodontal probing depth, “PPD”; bleeding on probing, “BoP”; gingival bleeding index, “GBI”; bleeding index, “BI”; gingival index, “GI”; modified gingival index, “MGI”; plaque index, “PI”; visible plaque index, “VPI”; modified plaque index, “MPI”; orthodontic plaque index, “OPI”; community periodontal index, “CPI”; papilla bleeding index, “PBI”; bonded bracket index, “BBI”; hyperplastic index, “HI”; colony forming units, “CFU”; mean difference, “MD”; standardized mean difference, “SMD”; weighted mean difference, “WMD”; prediction interval, “Pi”; confidence interval, “CI”; *p*-value, “*p*”.

**Table 2:** Synthesis of periodontal outcomes reported in the currently included systematic reviews related to the self-care intervention(s) investigated in periodontally healthy patients undergoing fixed orthodontic treatment. Evidence concerning manual and powered toothbrushes are in blue, chlorhexidine-containing products in yellow, other organic products in green, probiotics in fuchsia, and motivational methods in violet.

Authors, Year, Title	Methods and Comparison	Periodontal Outcomes (Statistically Significant)	Conclusions
ElShehaby, 2020 [42] Powered vs. manual tooth brushing in patients with fixed orthodontic appliances: A systematic review and meta-analysis	Manual toothbrush vs. powered toothbrush	At 4 and 8 w follow-up GI: NSS PI: NSS OPI: NSS	No differences in plaque or gingival index were found in fixed orthodontic patients using manual and powered toothbrushes at 4 and 8-week follow-ups
Fatima, 2020 [49] Effectiveness of antimicrobial gels on gingivitis during fixed orthodontic treatment: A systematic review and meta-analysis	Manual toothbrush with antimicrobial gels vs. manual toothbrush alone	At 2 w follow-up PPD: NNS At 4 w follow-up PPD: NNS	Antimicrobial gels in gingivitis management may improve periodontal health conditions in orthodontic patients No significant differences in PPD were detected between antimicrobial gel users and non-users at follow-ups
Pithon, 2017 [43] Effectiveness of different mechanical bacterial plaque removal methods in patients with the fixed orthodontic appliance: a systematic review and meta-analysis	Mechanical oral hygiene vs. different mechanical methods	Manual brush At 1 mo. follow-up PI: (MD: −1.01; 95% CI; −1.23 to −0.79) *p* < 0.001	Conventional manual toothbrushes were effective in reducing PI
Marçal, 2022 [45] Effectiveness of orthodontic toothbrush versus conventional toothbrush on plaque and gingival index reduction: A systematic review and meta-analysis	Orthodontic toothbrush vs. manual (conventional) toothbrush	GBI: NSS PI: (MD: −1.72; 95% CI; −0.83 to −2.61; 82% l^2^) *p* = 0.0001	Orthodontic toothbrushes do not modify gingival bleeding, but there is circumstantial scientific evidence for recommending the use of an orthodontic toothbrush instead of a conventional toothbrush for biofilm control
Al Makhmari, 2017 [54] Short-term and long-term effectiveness of powered toothbrushes in promoting periodontal health during orthodontic treatment: A systematic review and meta-analysis	Powered toothbrush vs. manual toothbrush	Short-term powered toothbrushes PPD: (WMD: −0.760; 95% CI; −1.029 to −0.491; n = 24) *p* = 0.000 GBI: (SMD: −0.637; 95% CI; −1.092 to −0.183; 95% Pi; −2.106 to −0.832; n = 342; l^2^ = 76%) *p* = 0.06 GI: (WMD: −0.079; 95% CI; −0.146 to −0.012; 95% Pi; −0.300 to 0.142; n = 374; l^2^ = 83%) *p* = 0.021 Long-term powered toothbrushes PPD: NSS GBI: (WMD: −1.630; 95% CI; −3.206 to −0.054; n = 40) *p* = 0.043 GI: (WMD: −0.220; 95% CI; −0.424 to −0.016; n = 40) *p* = 0.035	“Powered toothbrushes may benefit manual toothbrushes regarding gingival index and gingival bleeding assessments in orthodontic patients. However, no type demonstrated clear superiority”
Kaklamanos, 2008 [46] Meta-analysis on the effectiveness of powered toothbrushes for orthodontic patients	Powered toothbrush vs. manual toothbrush	BoP: NSS GI: NSS	No difference between manual or powered toothbrushing in fixed orthodontic patients were observed in the gingival index or bleeding scores
Hussain, 2022 [47] Effects of CHX use on periodontal health during fixed appliance orthodontic treatment: a systematic review and meta-analysis	CHX products (mouthwash, gel, toothpaste, varnishes) vs. no intervention OR placebo OR sodium fluoride products (mouthwash, gel, toothpaste, varnishes)	CHX mouthwash vs. placebo At 1 mo. follow-up GI: (MD: −0.67; 95% CI; −0.92 to −0.42; n = 3) *p* < 0.001 PI: (MD: −0.71; 95% CI; −0.90 to −0.52; n = 3) *p* < 0.001 At 3 mo. follow-up PPD: (MD: −0.60; 95% CI; −1.06 to −0.14; n = 2) *p* < 0.01 BI: (MD: −1.61; 95% CI; −2.99 to −0.22; n = 3) *p* < 0.02 GI: (MD: −0.68; 95% CI; −0.97 to −0.38; n = 9) *p* < 0.001 PI: (MD: −0.65; 95% CI; −0.86 to −0.43; n = 9) *p* < 0.001 At 6 mo. follow-up BI: (MD: −0.90; 95% CI; −1.39 to −0.40; n = 2) *p* < 0.001 GI: (MD: −0.44; 95% CI; −0.86 to −0.02; n = 2) *p* < 0.04 PI: NSS CHX gel vs. placebo At 1 mo. follow-up PPD: NSS At 3 mo. follow-up PPD: NSS GI: NSS PI: NSS CHX varnish vs. placebo At 3 mo. follow-up GI: NSS PI: NSS At 6 mo. follow-up GI: NNS PI: NSS CHX toothpaste vs. sodium fluoride mouthwash At 3 mo. follow-up BI: NSS GI: NSS OPI: (MD: −5.24; 95% CI; −10.46 to −0.02; n = 2) *p* < 0.04	CHX-containing mouthwashes were associated with lower GI, PI, BI, and PPD values in the short term No considerable benefits on GI, PI, or PPD were found from the use of CHX-gel or CHX-varnish The use of a CHX-containing toothpaste was more effective in lowering PI than the adjunct use of fluoride-containing mouthwash, but not GI or BI
Karamani, 2022 [48] CHX Mouthwash for Gingivitis Control in Orthodontic Patients: A Systematic Review and Meta-Analysis	CHX mouthwash vs. Any other mouthwash, including placebo solutions	GBI: N/D CHX vs. propolis/probiotics/herbs at 3 and 4 w: GI: NSS PI: N/D PBI: N/D BBI: N/D HI: N/D	“CHX mouthwash in orthodontic patients successfully controls gingival inflammation and bleeding when compared to untreated controls but is equally effective as other mouth rinses where various oral health indices are concerned”
Pithon, 2015 [44] Assessment of the effectiveness of mouthwashes in reducing cariogenic biofilm in orthodontic patients: a systematic review	Mouthwashes based on CHX, octenidine, essential oil, Cetylpyridinium, sodium fluoride, and amine fluoride/stannous fluoride vs. placebo mouthwash OR no intervention	PI: N/D	The orthodontists may suggest the use of oral antiseptics as adjunct in the PI reduction in periodontal self-care
Papadopoulou, 2021 [28] A systematic review on the effectiveness of organic unprocessed products in controlling gingivitis in patients undergoing orthodontic treatment with fixed appliances	Organic products (Aloe vera mouth rinse, ingestion of honey and chamomile mouthwash) vs. any other mouthwash, including placebo solutions	MTC vs. placebo Placebo group GBI: +23.1% VPI: +10.2% MTC group GBI: −29.9% VPI: −25.6% CHX group GBI: −32.0% PI: −31.39 ± 16.58 GI: −16.30 ± 9.98 VPI: −39.9% Aloe vera group PI: −20.38 ± 16.74 GI: 9.88 ± 8.77 Chlorine dioxide group: PI: −30.29 ± 18.30 GI: −12.22 ± 9.30 Gingival crevicular Bacterial counts: N/D Others pH: N/D	Non-pharmacological formulations reduced biofilm accumulation and gingival indices in orthodontic patients with gingivitis Their effect was attributed to their antimicrobial and anti-inflammatory activities No side effects similar to those associated with CHX were reported
Kommuri, 2022 [29] Efficacy of herbal- versus CHX-based mouthwashes towards oral hygiene maintenance in patients undergoing fixed orthodontic therapy: A systematic review and meta-analysis	Organic products (herbal-based mouthwash) vs. CHX-based mouthwash	PPD: N/D BoP: N/D GI: N/D PI: N/D CPI: N/D HI: N/D Others CFU of oral bacteria: N/D	The comparison between the efficacy of herbal and CHX mouthwashes on biofilm control and inflammation reversal remains debatable Three studies found that the CHX-based mouthwashes were superior, while four studies showed that CHX was as effective as herbal-based mouthwashes
Panagiotou, 2021 [27] Role of Essential Oil-Based Mouthwashes in Controlling Gingivitis in Patients Undergoing Fixed Orthodontic Treatment: A Review of Clinical Trials	Essential oil mouthwash (Listerine^®^, Listerine Fructus mume, MTC, Zingiber officinale) vs. mouthwashes not containing essential oils (CHX, povidone-iodine, placebo, distilled water) OR no mouthwash	GBI: N/D BI: N/D MGI: N/D PI: N/D VPI: N/D MPI: N/D	Essential oil-based mouthwashes seem to be effective in gingivitis management in subjects undergoing fixed orthodontic treatment
Pietri, 2020 [50] Role of Probiotics in Oral Health Maintenance Among Patients Undergoing Fixed Orthodontic Therapy: a Systematic Review of Randomized Controlled Clinical Trials	Probiotics vs. no probiotics	GI: N/D PI: N/D Others Subgingival levels of *Porphyromonas gingivalis*: N/D Salivary streptococcal colony count: N/D *Streptococcus mutans* and *Lactobacillus* scores in plaque and saliva: N7D Halitosis: N/D	Probiotics exhibit antimicrobial activity against oral pathogenic bacteria (S.Mutans and Lactobacillus), decreasing their counts in saliva and biofilm
Hadj-Hamou, 2020 [51] Do probiotics promote oral health during orthodontic treatment with fixed appliances? A systematic review	Probiotics vs. placebo OR no intervention	GI: NSS	Probiotic administration does not seem to have an effect on gingival inflammation
Huang, 2018 [34] Effects of motivational methods on oral hygiene of orthodontic patients: A systematic review and meta-analysis	Motivational methods vs. different motivational methods OR no motivational methods	At 1 mo. follow-up GI: (MD: −017; Cl 95%; −0.23 to −0.11) *p* < 0.05 PI: NSS At 3 mo. follow-up GI: (MD: −0.20; CI 95%; −0.33 to −0.06) *p* < 0.05 PI: (MD: −0.23; Cl 95%; −0.39 to −0.06) *p* < 0.05 At 6-mo. follow-up GI: (MD: −0.30; Cl 95%; −0.36 to −0.23) *p* < 0.05 PI: (MD: −0.19; Cl 95%; −0.35 to −0.03) *p* < 0.05	A motivational method, or ideally their combination, can improve biofilm control Reinforcement during the orthodontic treatment period is useful
Sharif, 2019 [52] A systematic review to assess interventions delivered by mobile phones in improving adherence to oral hygiene advice for children and adolescents	Any interventions delivered by mobile phones vs. any interventions delivered not using mobile phones	Text messages group GI: N/D PI: N/D Mobile phone app and smartphone video tutorials group At 3 mo. GBI: NSS PI: NSS At 6 mo. GBI: *p* < 0.01 PI: *p* < 0.01 At 9 mo. GBI: *p* < 0.05 PI: *p* < 0.01 At 12 mo. GBI: *p* < 0.05 PI: *p* < 0.01	Some evidence suggests that mobile phones are effective in improving adherence to oral hygiene procedures in orthodontic patients
Migliorati, 2015 [53] Efficacy of professional hygiene and prophylaxis on preventing plaque increase in orthodontic patients with multibracket appliances: a systematic review	Motivational methods, professional hygiene, and prophylaxis regimen vs. usual self-care OR no intervention	GI: N/D PI: N/D	Regular patient motivation sessions and professional mechanical supragingival biofilm removal help maintain good biofilm control during fixed orthodontic treatment

Abbreviations: month(s), “mo.”; week(s), “w”; not defined, “N/D”; not statistically significant, “NNS”; Chlorhexidine, “CHX”; Matricaria chamomilla L, “MTC”; periodontal probing depth, “PPD”; bleeding on probing, “BoP”; gingival bleeding index, “GBI”; bleeding index, “BI”; gingival index, “GI”; modified gingival index, “MGI”; plaque index, “PI”; visible plaque index, “VPI”; modified plaque index, “MPI”; orthodontic plaque index, “OPI”; community periodontal index, “CPI”; papilla bleeding index, “PBI”; bonded bracket index, “BBI”; hyperplastic index, “HI”; colony forming units, “CFU”; mean difference, “MD”; standardized mean difference, “SMD”; weighted mean difference, “WMD”; prediction interval, “Pi”; confidence interval, “CI”; *p*-value, “*p*”.

## Data Availability

Data supporting the reported results can be found in the PROSPERO Registry and the Cochrane Library, Web of Science (Core Collection), Scopus, and MEDLINE/PubMed databases.

## References

[1] Lang N.P., Bartold P.M. (2018). Periodontal Health. J. Periodontol..

[2] Tonetti M.S., Eickholz P., Loos B.G., Papapanou P., van der Velden U., Armitage G., Bouchard P., Deinzer R., Dietrich T., Hughes F. (2015). Principles in Prevention of Periodontal Diseases. J. Clin. Periodontol..

[3] Martina S., Martini M., Bordegoni M., Razionale A.V. (2021). Predictability of Root Movements Using Virtual Root Setup in a Patient With Periodontal Disease Treated With Clear Aligners. Open Dent. J..

[4] Giuca M.R., Pasini M., Drago S., del Corso L., Vanni A., Carli E., Manni A. (2020). Influence of Vertical Facial Growth Pattern on Herbst Appliance Effects in Prepubertal Patients: A Retrospective Controlled Study. Int. J. Dent..

[5] Heintze S.D., Jost-Brinkman P., Finke C., Miethke R.R. (1999). Oral Health for the Orthodontic Patient.

[6] Megha S., Shalini G., Varsha S.A., Abhishek D., Neetu J. (2019). Effect of Short-Term Placebo-Controlled Consumption of Probiotic Yoghurt and Indian Curd on the Streptococcus Mutans Level in Children Undergoing Fixed Interceptive Orthodontic Therapy. Turk. J. Orthod..

[7] Carli E., Pasini M., Lardani L., Giuca G., Miceli M. (2021). Impact of Self-Ligating Orthodontic Brackets on Dental Biofilm and Periodontal Pathogens in Adolescents. J. Biol. Regul. Homeost. Agents.

[8] Karkhanechi M., Chow D., Sipkin J., Sherman D., Boylan R.J., Norman R.G., Craig R.G., Cisneros G.J. (2013). Periodontal Status of Adult Patients Treated with Fixed Buccal Appliances and Removable Aligners over One Year of Active Orthodontic Therapy. Angle Orthod..

[9] Amato A. (2022). Oral-Systemic Health and Disorders: Latest Advances on Oral–Gut–Lung Microbiome Axis. Appl. Sci..

[10] Mummolo S., Nota A., Albani F., Marchetti E., Gatto R., Marzo G., Quinzi V., Tecco S. (2020). Salivary Levels of Streptococcus Mutans and Lactobacilli and Other Salivary Indices in Patients Wearing Clear Aligners versus Fixed Orthodontic Appliances: An Observational Study. PLoS ONE.

[11] D’ambrosio F., Caggiano M., Schiavo L., Savarese G., Carpinelli L., Amato A., Iandolo A. (2022). Chronic Stress and Depression in Periodontitis and Peri-Implantitis: A Narrative Reviewon Neurobiological, Neurobehavioral and Immune–Microbiome Interplays and Clinical Management Implications. Dent. J..

[12] Wennström J.L., Stokland B.L., Nyman S., Thilander B. (1993). Periodontal Tissue Response to Orthodontic Movement of Teeth with Infrabony Pockets. Am. J. Orthod. Dentofac. Orthop..

[13] di Spirito F., Toti P., Brevi B., Martuscelli R., Sbordone L., Sbordone C. (2019). Computed tomography evaluation of jaw atrophies before and after surgical bone augmentation. Int. J. Clin. Dent..

[14] Martin C., Celis B., Ambrosio N., Bollain J., Antonoglou G.N., Figuero E. (2022). Effect of Orthodontic Therapy in Periodontitis and Non-periodontitis Patients: A Systematic Review with Meta-analysis. J. Clin. Periodontol..

[15] van Gastel J., Quirynen M., Teughels W., Coucke W., Carels C. (2011). Longitudinal Changes in Microbiology and Clinical Periodontal Parameters after Removal of Fixed Orthodontic Appliances. Eur. J. Orthod..

[16] Papageorgiou S.N., Eliades T., Eliades T., Katsaros C. (2019). Clinical Evidence on the Effect of Orthodontic Treatment on the Periodontal Tissues. The Ortho-Perio Patient: Clinical Evidence & Therapeutic Guidelines.

[17] Gomes S.C., Varela C.C., da Veiga S.L., Rosing C.K., Oppermann R.V. (2007). Periodontal Conditions in Subjects Following Orthodontic Therapy. A Preliminary Study. Eur. J. Orthod..

[18] Pace M., Cioffi I., D’antò V., Valletta A., Valletta R., Amato M. (2018). Facial Attractiveness of Skeletal Class i and Class II Malocclusion as Perceived by Laypeople, Patients and Clinicians. Minerva Stomatol..

[19] Rongo R., Bucci R., Adaimo R., Amato M., Martina S., Valletta R., D’antò V. (2020). Two-Dimensional versus Three-Dimensional Fränkel Manoeuvre: A Reproducibility Study. Eur J. Orthod.

[20] di Spirito F. (2022). Oral-Systemic Health and Disorders: Latest Prospects on Oral Antisepsis. Appl. Sci..

[21] Giuca M.R., Lardani L., Ligori S., Carli E., Giuca G., Miceli M. (2021). Oral Manifestations in Paediatric Patients with Hepatobiliary Diseases: A Review. J. Biol. Regul. Homeost. Agents.

[22] Albertsson K.W., van Dijken J.W. (2010). Awareness of Toothbrushing and Dentifrice Habits in Regularly Dental Care Receiving Adults. Swed. Dent. J..

[23] Graziani F., Karapetsa D., Alonso B., Herrera D. (2017). Nonsurgical and Surgical Treatment of Periodontitis: How Many Options for One Disease?. Periodontol. 2000.

[24] Arici S., Alkan A., Arici N. (2007). Comparison of Different Toothbrushing Protocols in Poor-Toothbrushing Orthodontic Patients. Eur. J. Orthod..

[25] Boccia G., Di Spirito F., D’Ambrosio F., Di Palo M.P., Giordano F., Amato M. (2023). Local and systemic antibiotics in peri-implantitis management: An umbrella review. Antibiotics.

[26] Löe H., Schiott C.R. (1970). The Effect of Mouthrinses and Topical Application of Chlorhexidine on the Development of Dental Plaque and Gingivitis in Man. J. Periodontal Res..

[27] Panagiotou A., Rossouw P.E., Michelogiannakis D., Javed F. (2021). Role of Essential Oil-Based Mouthwashes in Controlling Gingivitis in Patients Undergoing Fixed Orthodontic Treatment. A Review of Clinical Trials. Int. J. Env. Res. Public Health.

[28] Papadopoulou C., Karamani I., Gkourtsogianni S., Seremidi K., Kloukos D. (2021). A Systematic Review on the Effectiveness of Organic Unprocessed Products in Controlling Gingivitis in Patients Undergoing Orthodontic Treatment with Fixed Appliances. Clin. Exp. Dent. Res..

[29] Kommuri K., Michelogiannakis D., Barmak B.A., Rossouw P.E., Javed F. (2022). Efficacy of Herbal-versus Chlorhexidine-based Mouthwashes towards Oral Hygiene Maintenance in Patients Undergoing Fixed Orthodontic Therapy: A Systematic Review and Meta-analysis. Int. J. Dent. Hyg..

[30] World Health Organization (2006). Probiotics in food. Health and Nutritional Properties and Guidelines for Evaluation.

[31] Ikram S., Hassan N., Baig S., Borges K.J.J., Raffat M.A., Akram Z. (2019). Effect of Local Probiotic (*Lactobacillus reuteri*) vs Systemic Antibiotic Therapy as an Adjunct to Non-surgical Periodontal Treatment in Chronic Periodontitis. J. Investig. Clin. Dent..

[32] Costacurta M., Sicuro L., Margiotta S., Ingrasciotta I. (2018). Clinical Effects of Lactobacillus Reuteri Probiotic in Treatment of Chronic Periodontitis. A Randomized, Controlled Trial. Oral Implant..

[33] Amato M., di Spirito F., D’Ambrosio F., Boccia G., Moccia G., de Caro F. (2022). Probiotics in Periodontal and Peri-Implant Health Management: Biofilm Control, Dysbiosis Reversal, and Host Modulation. Microorganisms.

[34] Huang J., Yao Y., Jiang J., Li C. (2018). Effects of Motivational Methods on Oral Hygiene of Orthodontic Patients: A Systematic Review and Meta-Analysis. Medicine.

[35] Bifulco M., Amato M., Gangemi G., Marasco M., Caggiano M., Amato A., Pisanti S. (2016). Dental care and dentistry practice in the medieval medical school of salerno. Br. Dent. J..

[36] Page M.J., McKenzie J.E., Bossuyt P.M., Boutron I., Hoffmann T.C., Mulrow C.D., Shamseer L., Tetzlaff J.M., Akl E.A., Brennan S.E. (2021). The PRISMA 2020 Statement: An Updated Guideline for Reporting Systematic Reviews. Int. J. Surg..

[37] Higgins J.P.T., Thomas J., Chandler J., Cumpston M., Li T., Page M.J., Welch V. (2022). Cochrane Handbook for Systematic Reviews of Interventions Version 6.3 (Updated February 2022). Cochrane. www.training.cochrane.org/handbook.

[38] Richardson W.S., Wilson M.C., Nishikawa J., Hayward R.S. (1995). The Well-Built Clinical Question: A Key to Evidence-Based Decisions. ACP J. Club.

[39] Di Spirito F., Argentino S., Martuscelli R., Sbordone L. (2019). Mronj incidence after multiple teeth extractions in patients taking oral bis-phosphonates without “drug holiday”: A retrospective chart review. Oral Implantol.

[40] di Spirito F., Caggiano M., di Palo M.P., Contaldo M., D’Ambrosio F., Martina S., Amato A. (2022). Oral Lesions in Pediatric Subjects: SARS-CoV-2 Infection and COVID-19 Vaccination. Appl. Sci..

[41] Shea B.J., Reeves B.C., Wells G., Thuku M., Hamel C., Moran J., Moher D., Tugwell P., Welch V., Kristjansson E. (2017). AMSTAR 2: A Critical Appraisal Tool for Systematic Reviews That Include Randomised or Non-Randomised Studies of Healthcare Interventions, or Both. BMJ.

[42] ElShehaby M., Mofti B., Montasser M.A., Bearn D. (2020). Powered vs Manual Tooth Brushing in Patients with Fixed Orthodontic Appliances: A Systematic Review and Meta-Analysis. Am. J. Orthod. Dentofac. Orthop..

[43] Pithon M.M., Sant’Anna L.I.D.A., Baião F.C.S., Coqueiro R.D.S., Maia L.C., Paranhos L.R. (2017). Effectiveness of Different Mechanical Bacterial Plaque Removal Methods in Patients with Fixed Orthodontic Appliance: A Systematic Review/Meta-Analysis. Biosci. J..

[44] Pithon M.M., Sant’Anna L.I.D.A., Baião F.C.S., dos Santos R.L., Coqueiro R.d.S., Maia L.C. (2015). Assessment of the Effectiveness of Mouthwashes in Reducing Cariogenic Biofilm in Orthodontic Patients: A Systematic Review. J. Dent..

[45] Marçal F.F., Mota de Paulo J.P., Barreto L.G., de Carvalho Guerra L.M., Silva P.G.d.B. (2022). Effectiveness of Orthodontic Toothbrush versus Conventional Toothbrush on Plaque and Gingival Index Reduction: A Systematic Review and Meta-Analysis. Int. J. Dent. Hyg..

[46] Kaklamanos E.G., Kalfas S. (2008). Meta-Analysis on the Effectiveness of Powered Toothbrushes for Orthodontic Patients. Am. J. Orthod. Dentofac. Orthop..

[47] Hussain U., Alam S., Rehman K., Antonoglou G.N., Papageorgiou S.N. (2022). Effects of Chlorhexidine Use on Periodontal Health during Fixed Appliance Orthodontic Treatment: A Systematic Review and Meta-Analysis. Eur. J. Orthod..

[48] Karamani I., Kalimeri E., Seremidi K., Gkourtsogianni S., Kloukos D. (2022). Chlorhexidine Mouthwash for Gingivitis Control in Orthodontic Patients: A Systematic Review and Meta-Analysis. Oral Health Prev. Dent..

[49] Fatima F., Taha Mahmood H., Fida M., Hoshang Sukhia R. (2020). Effectiveness of Antimicrobial Gels on Gingivitis during Fixed Orthodontic Treatment: A Systematic Review and Meta-Analysis. Int. Orthod..

[50] Pietri F.K., Rossouw P.E., Javed F., Michelogiannakis D. (2020). Role of Probiotics in Oral Health Maintenance Among Patients Undergoing Fixed Orthodontic Therapy: A Systematic Review of Randomized Controlled Clinical Trials. Probiotics Antimicrob Proteins.

[51] Hadj-Hamou R., Senok A.C., Athanasiou A.E., Kaklamanos E.G. (2020). Do Probiotics Promote Oral Health during Orthodontic Treatment with Fixed Appliances? A Systematic Review. BMC Oral Health.

[52] Sharif M.O., Newton T., Cunningham S.J. (2019). A Systematic Review to Assess Interventions Delivered by Mobile Phones in Improving Adherence to Oral Hygiene Advice for Children and Adolescents. Br. Dent. J..

[53] Migliorati M., Isaia L., Cassaro A., Rivetti A., Silvestrini-Biavati F., Gastaldo L., Piccardo I., Dalessandri D., Silvestrini-Biavati A. (2015). Efficacy of Professional Hygiene and Prophylaxis on Preventing Plaque Increase in Orthodontic Patients with Multibracket Appliances: A Systematic Review. Eur. J. Orthod..

[54] al Makhmari S.A., Kaklamanos E.G., Athanasiou A.E. (2017). Short-Term and Long-Term Effectiveness of Powered Toothbrushes in Promoting Periodontal Health during Orthodontic Treatment: A Systematic Review and Meta-Analysis. Am. J. Orthod. Dentofac. Orthop..

[55] Proffit W.R., Fields H.W., Sarver D.M. (2013). Ortodonzia Moderna.

[56] Billings M., Holtfreter B., Papapanou P.N., Mitnik G.L., Kocher T., Dye B.A. (2018). Age-Dependent Distribution of Periodontitis in Two Countries: Findings from NHANES 2009 to 2014 and SHIP-TREND 2008 to 2012. J. Periodontol..

[57] Kelsey J.L., Lamster I.B. (2008). Influence of Musculoskeletal Conditions on Oral Health Among Older Adults. Am. J. Public Health.

[58] Waldron C., Nunn J., mac Giolla Phadraig C., Comiskey C., Guerin S., van Harten M.T., Donnelly-Swift E., Clarke M.J. (2019). Oral Hygiene Interventions for People with Intellectual Disabilities. Cochrane Database Syst. Rev..

[59] Silva A.M., Miranda L.F.B., Araújo A.S.M., Prado Júnior R.R., Mendes R.F. (2020). Electric Toothbrush for Biofilm Control in Individuals with Down Syndrome: A Crossover Randomized Clinical Trial. Braz. Oral Res..

[60] Albandar J.M., Susin C., Hughes F.J. (2018). Manifestations of Systemic Diseases and Conditions That Affect the Periodontal Attachment Apparatus: Case Definitions and Diagnostic Considerations. J. Clin. Periodontol..

[61] Ren Y., Jongsma M.A., Mei L., van der Mei H.C., Busscher H.J. (2014). Orthodontic Treatment with Fixed Appliances and Biofilm Formation—A Potential Public Health Threat?. Clin. Oral Investig..

[62] Johnson B.D., McLnnes C. (1994). Clinical Evaluation of the Efficacy and Safety of a New Sonic Toothbrush. J. Periodontol..

[63] Harnacke D., Beldoch M., Bohn G.-H., Seghaoui O., Hegel N., Deinzer R. (2012). Oral and Written Instruction of Oral Hygiene: A Randomized Trial. J. Periodontol..

[64] Deinzer R., Ebel S., Blättermann H., Weik U., Margraf-Stiksrud J. (2018). Toothbrushing: To the Best of One’s Abilities Is Possibly Not Good Enough. BMC Oral Health.

[65] Deinzer R., Schmidt R., Harnacke D., Meyle J., Ziebolz D., Hoffmann T., Wöstmann B. (2018). Finding an Upper Limit of What Might Be Achievable by Patients: Oral Cleanliness in Dental Professionals after Self-Performed Manual Oral Hygiene. Clin. Oral Investig..

[66] Introducing the Oral-B GENIUS Brush | Oral-B—YouTube. https://www.youtube.com/watch?v=9UdUM7Q_Pw8.

[67] Yaacob M., Worthington H.V., Deacon S.A., Deery C., Walmsley A.D., Robinson P.G., Glenny A.-M. (2014). Powered versus Manual Toothbrushing for Oral Health. Cochrane Database Syst. Rev..

[68] Leikin B.J., Paloucek F.P. (2008). Chlorhexidine Gluconate. Poisoning and Toxicology Handbook.

[69] EMILSON C.G. (1977). Susceptibility of Various Microorganisms to Chlorhexidine. Eur. J. Oral Sci..

[70] Sanz M., Herrera D., Kebschull M., Chapple I., Jepsen S., Berglundh T., Sculean A., Tonetti M.S., Merete Aass A., Aimetti M. (2020). Treatment of Stage I–III Periodontitis—The EFP S3 Level Clinical Practice Guideline. J. Clin. Periodontol..

[71] da Costa L.F.N.P., da Silva Furtado Amaral C., da Silva Barbirato D., Leão A.T.T., Fogacci M.F. (2017). Chlorhexidine Mouthwash as an Adjunct to Mechanical Therapy in Chronic Periodontitis. J. Am. Dent. Assoc..

[72] Pisano M., Amato A., Sammartino P., Iandolo A., Martina S., Caggiano M. (2021). Laser therapy in the treatment of peri-implantitis: State-of-the-art, literature review and meta-analysis. Appl. Sci..

[73] Hamilton I.R. (1990). Biochemical Effects of Fluoride on Oral Bacteria. J. Dent. Res..

[74] Bowden G.H.W. (1990). Effects of Fluoride on the Microbial Ecology of Dental Plaque. J. Dent. Res..

[75] Waerhaug J. (1981). Effect of Toothbrushing on Subgingival Plaque Formation. J. Periodontol..

[76] James P., Worthington H.V., Parnell C., Harding M., Lamont T., Cheung A., Whelton H., Riley P. (2017). Chlorhexidine Mouthrinse as an Adjunctive Treatment for Gingival Health. Cochrane Database Syst. Rev..

[77] Ouderaa F.J.G. (1991). Anti-Plaque Agents. Rationale and Prospects for Prevention of Gingivitis and Periodontal Disease. J. Clin. Periodontol..

[78] Goes P., Dutra C.S., Lisboa M.R.P., Gondim D.V., Leitão R., Brito G.A.C., Rego R.O. (2016). Clinical Efficacy of a Matricaria Chamomile. Mouthwash and 0.12% Chlorhexidine for Gingivitis Control in Patients Undergoing Orthodontic Treatment with Fixed Appliances. J. Oral Sci..

[79] Kochhar S.L. (2009). Economic Botany in the Tropics.

[80] Bauer Faria T.R., Furletti-Goes V.F., Franzini C.M., de Aro A.A., de Andrade T.A.M., Sartoratto A., de Menezes C.C. (2021). Anti-Inflammatory and Antimicrobial Effects of Zingiber Officinale Mouthwash on Patients with Fixed Orthodontic Appliances. Am. J. Orthod. Dentofac. Orthop..

[81] Chen M.S., Andersen R.M., Barmes D.E., Leclerq M.H., Lyttle C.S. (1997). ; World Health Organization. Comparing Oral Health Care Systems: A Second International Collaborative Study.

[82] Yeturu S.K., Acharya S., Urala A.S., Pentapati K.C. (2016). Effect of Aloe Vera, Chlorine Dioxide, and Chlorhexidine Mouth Rinses on Plaque and Gingivitis: A Randomized Controlled Trial. J. Oral Biol. Craniofac. Res..

[83] Meurman J., Stamatova I. (2007). Probiotics: Contributions to Oral Health. Oral Dis..

[84] Twetman S., Keller M.K. (2012). Probiotics for Caries Prevention and Control. Adv. Dent. Res..

[85] Gruner D., Paris S., Schwendicke F. (2016). Probiotics for Managing Caries and Periodontitis: Systematic Review and Meta-Analysis. J. Dent..

[86] D’Ambrosio F., Pisano M., Amato A., Iandolo A., Caggiano M., Martina S. (2022). Periodontal and peri-implant health status in traditional vs. heat-not-burn tobacco and electronic cigarettes smokers: A systematic review. Dent. J..

[87] Al-Jewair T.S., Suri S., Tompson B.D. (2011). Predictors of Adolescent Compliance with Oral Hygiene Instructions during Two-Arch Multibracket Fixed Orthodontic Treatment. Angle Orthod..

[88] Cozzani M., Ragazzini G., Delucchi A., Mutinelli S., Barreca C., Rinchuse D.J., Servetto R., Piras V. (2016). Oral Hygiene Compliance in Orthodontic Patients: A Randomized Controlled Study on the Effects of a Post-Treatment Communication. Prog. Orthod..

[89] Kay E., Locker D. (1998). A Systematic Review of the Effectiveness of Health Promotion Aimed at Improving Oral Health. Community Dent. Health.

[90] di Spirito F., Amato A., di Palo M.P., Ferraro G.A., Baroni A., Serpico R., Contaldo M. (2022). COVID-19 related information on pediatric dental care including the use of teledentistry: A narrative review. Children.

[91] Amato A., Iandolo A., Scelza G., Spirito F., Martina S. (2022). COVID-19: The Patients’ Perceived Impact on Dental Care. Eur. J. Dent..

[92] Reddy M., Shetty S., Vannala V. (2019). Embracing Personalized Medicine in Dentistry. J. Pharm. Bioallied Sci..

[93] di Spirito F. (2023). Integrating P4 Medicine in Teledentistry and M-Health in Oral, Dental, and Periodontal Care. J. Pers. Med..

[94] Li X., Xu Z.-R., Tang N., Ye C., Zhu X.-L., Zhou T., Zhao Z.-H. (2016). Effect of Intervention Using a Messaging App on Compliance and Duration of Treatment in Orthodontic Patients. Clin. Oral Investig..

[95] Brent Bowen T., Rinchuse D.J., Zullo T., DeMaria M.E. (2015). The Influence of Text Messaging on Oral Hygiene Effectiveness. Angle Orthod..

[96] Amato M., Zingone F., Caggiano M., Iovino P., Bucci C., Ciacci C. (2017). Tooth wear is frequent in adult patients with celiac disease. Nutrients.

[97] Amato A., Ciacci C., Martina S., Caggiano M., Amato M. (2021). COVID-19: The dentists’ perceived impact on the dental practice. Eur. J. Dent..

[98] Poppolo Deus F., Ouanounou A. (2022). Chlorhexidine in Dentistry: Pharmacology, Uses, and Adverse Effects. Int. Dent. J..

[99] Chye R.M.L., Perrotti V., Piattelli A., Iaculli F., Quaranta A. (2019). Effectiveness of Different Commercial Chlorhexidine-Based Mouthwashes After Periodontal and Implant Surgery. Implant. Dent..

